# Targeting the pro-inflammatory factor CCL2 (MCP-1) with Bindarit for influenza A (H7N9) treatment

**DOI:** 10.1038/cti.2017.8

**Published:** 2017-03-31

**Authors:** Stefan Wolf, Scott Johnson, Olivia Perwitasari, Suresh Mahalingam, Ralph A Tripp

**Affiliations:** 1 Department of Infectious Diseases, University of Georgia, Athens, GA, USA; 2 Institute for Glycomics, Griffith University, Gold Coast Campus, Queensland, Australia

## Abstract

Influenza A viruses are important human and animal pathogens. Seasonal influenza viruses cause infections every year, and occasionally zoonotic viruses emerge to cause pandemics with significantly higher morbidity and mortality rates. Three cases of laboratory confirmed human infection with avian influenza A (H7N9) virus were reported in 2013, and there have been several cases reported across South East Asia, and recently in North America. Most patients experience severe respiratory illness, with mortality rates approaching 40%. No vaccine is currently available and the use of antivirals is complicated due to the emergence of drug resistant strains. Thus, there is a need to identify new drugs for therapeutic intervention and disease control. In humans, following H7N9 infection, there is excessive expression of pro-inflammatory factors CCL2, IL-6, IL-8, IFNα, interferon-γ, IP-10, MIG and macrophage inflammatory protein-1β, which has been shown to contribute to fatal disease outcomes in mouse models of infection. In the current study, the potent inhibitor of CCL2 synthesis, Bindarit, was examined as a countermeasure for H7N9-induced inflammation in a mouse model. Bindarit treatment of mice did not have any substantial therapeutic efficacy in H7N9 infection. Consequently, the results suggest that Bindarit may be ill-advised in the treatment of influenza H7N9 infection.

Despite vaccines and therapeutics for circulating strains, influenza A virus (IAV) remains a serious global health threat affecting humans, wildlife and agricultural species. Avian IAV H7N9 virus (H7N9) infections in humans were first reported in China in March 2013.^1^ Most infections are believed to have resulted from exposure to infected poultry or contaminated environments, as H7N9 viruses have been found in poultry in China. Infected children typically suffer only mild disease,^2^ whereas elderly patients are generally more severely afflicted.^3^ This suggests that there is minimal heterologous immunity from previous influenza infections. Most patients infected with H7N9 experienced severe respiratory illness, such as pneumonia (97.3%) and acute respiratory distress syndrome (71.2%), leading to high rates of intensive care unit admissions.^4^ The mortality rate attributed to influenza H7N9 infection in human is >38%, with 175 deaths from 450 confirmed cases reported within a 20-month period.^5^ No evidence of sustained human-to-human transmission of H7N9 has been recorded. However, there is some evidence of limited person-to-person spread under rare circumstances.^6^ After human H7N9 infections were first reported in China, the virus spread rapidly to other countries.^7^ Recently, the first case of human H7N9 infected in North America was documented in Canada.^8^

No specific vaccine is currently available for H7N9.^9^ There are several antivirals available for the treatment of IAV infections including the M2 ion channel inhibitors amantadine and rimantadine, and the neuraminidase inhibitors zanamivir and oseltamivir.^10, 11^ Early treatment with these antiviral drugs reduces the duration of symptoms and recovery time. However, the use of antiviral drugs is complicated by the emergence of drug resistant viruses,^12, 13^ and oseltamivir-resistant H7N9 strains have recently been described in Taiwan.^14^ In addition, the use of antiviral drugs may affect population vulnerability due to lack of seroconversion, as well as driving drug resistance among circulating strains.^15^ The development of new drugs and vaccines against H7N9 may take many years. Drug repurposing, or the use of clinical drug candidates, may help to overcome this lengthy process of drug development and reduce the impact of H7N9-induced disease. H7N9 disease results in a potent immune response believed to contribute to tissue destruction and pathology.^16^ The host's immune response towards H7N9 infection has not been fully characterized, which is an issue for the discovery of novel therapeutics and target identification for drug repurposing.

In studies of fatal H7N9 infections in humans, there is evidence of immune pathological changes caused by a heightened innate immune response.^16^ Proliferation of H7N9 in the lower respiratory tract causes excessive activation of the innate immune response.^16^ This leads to production of inflammatory mediators, many expressed by intrapulmonary macrophages and alveolar cells.^16^ Some of the major histological characteristics of H7N9 infection include diffuse alveolar damage, hyaline membrane and fibroproliferation in the lung, and spotty necrosis in the liver.^16^ Histological observations have shown a depletion of T cells, a fluctuating numbers of neutrophils, and highly abundant and activated macrophages which are characteristic of H7N9 infection in the alveoli.^16^ In addition, high levels of intrapulmonary inflammatory mediators, such as the interferon gamma-induced protein 10 (IP-10) and interleukin-6 (IL-6) have been detected.^16^ Plasma levels of IL-6 and IL-8 are sharply upregulated, whereas IL-10, macrophage inflammatory protein-1β, and interferon-γ are increased to intermediate levels.^4^ In contrast, IL-1β, tumor necrosis factor α (TNFα), and MIP-1α are found only at minimal concentrations. Whether the elevated cytokine and chemokine levels cause or contribute to the severity of H7N9 disease has yet to be determined.^4^ However, the upregulation of pro-inflammatory cytokines and chemokines, such as the monocyte chemoattractant protein-1 (MCP-1/CCL2), IL-6, IL-8, IFN-α, IP-10, MIG and macrophage inflammatory protein-1β was described in H7N9-infected patients with lung injury and severe pneumonia.^2^ The cytokine levels in C57BL/6 and BALB/c mice infected with H7N9 (A/Anhui/A/2013 strain) were compared. C57BL/6 mice exhibited more severe lung injury, slower recovery from lung damage, less effective viral clearance, higher levels of CCL2, IL-6 and IL1β, and lower levels of TNFα and interferon-γ than BALB/c mice. These data suggest that TNFα and IFNγ may help to suppress viral gene expression and increase viral clearance, while CCL2 and IL-6 may contribute to lung injury during H7N9 disease.^17^

The focus of this study was to assess the effects of drug inhibition of the pro-inflammatory factor CCL2, which may have a pathogenic role during H7N9 disease. Bindarit represents a novel class of inhibitor that reduces CCL2 synthesis.^18^ Bindarit has been successfully used to alleviate virus-induced inflammation in several animal models of disease. For example, Bindarit was efficacious in mouse models of Chikungunya and Ross River virus infections, where it was shown to ameliorate infections and disease.^19, 20^ Furthermore, Bindarit was able to reduce arthritic inflammation without showing any detrimental effect on virus clearance in these animal models of alphavirus infection.^21^ Therefore, the activity of Bindarit activity was determined to reduce pulmonary and serum CCL2 levels in a mouse model of H7N9 disease. Bindarit selectively inhibits CCL2. CCL2 is a critical mediator of neuroinflammation in myriad disease states, including multiple sclerosis,^22^ human immunodeficiency virus (HIV)-1-induced encephalitis,^23^ Guillain-Barré syndrome,^24^ Alzheimer's disease,^25^ ischemia,^26^ neurotrauma,^27^ epilepsy,^28^ neurogenic hypertension^29^ and alcoholism.^30^ Bindarit has also been studied for therapeutic intervention for these diseases. The clinical tolerability data for Bindarit in different CCL2-dependent illnesses demonstrated Bindarit safety up to a maximum dose of 2400 mg per day for as long as 6 months, and suggests the potential of Bindarit to be beneficial for a range of diseases.^31^

This study aimed to explore the role of CCL2 in H7N9 disease and the potential of Bindarit to act as a countermeasure against H7N9-induced pathology in a mouse model. Intriguingly, the survival rate of Bindarit-treated mice was comparable to that of non-treated mice, while weight loss, cellular infiltration and viral titers were considerably increased with Bindarit treatment. Thus, the use of Bindarit as a therapeutic to treat H7N9 disease seems ill-advised.

## Results

### Bindarit treatment reduces *CCL2* gene expression in lung epithelial cells after IAV infection

**Figure 1:**
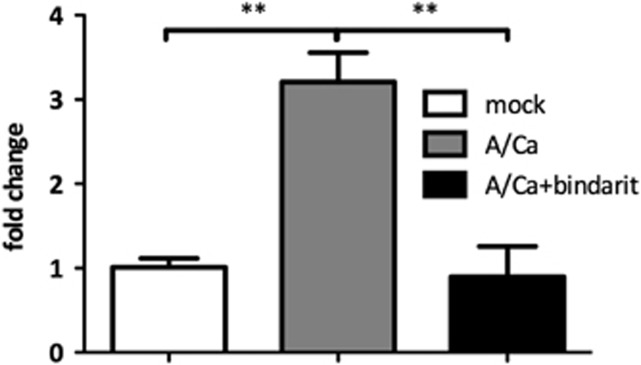
Bindarit reduces CCL2 gene expression in IAV-infected cells. A549 cells were infected with A/Ca (H1N1) at MOI 0.1 and subsequently treated with Bindarit at 100 μm. At 24 h pi, RNA was extracted for host gene expression analysis of *CCL2* using RT-qPCR. Expression was normalized to 18 s and compared to non-infected cells. Data are from two independent experiments using three replicates per group.

To evaluate the effectiveness of Bindarit in reducing CCL2 production, lung epithelial cells were infected (MOI=0.1) with A/California/04/09 (A/Ca; H1N1) virus and simultaneously treated with Bindarit. Quantitative reverse transcription PCR (RT-qPCR) was used to investigate the effect of Bindarit on *CCL2* gene expression during infection with A/Ca (H1N1), a representative, currently circulating IAV subtype, in a human epithelial cell (A549) line. CCL2 was considerably upregulated by 3.21-fold (*P*<0.01) in A/Ca (H1N1)-infected A549 cells compared to mock-infected controls at 24 h pi (Figure 1). When A549 cells were treated with Bindarit (100 μm), *CCL2* gene expression was significantly (*P*<0.01) reduced to a level comparable to mock-infected controls.

### Bindarit treatment does not protect mice from lethal avian IAV H7N9 infection

**Figure 2:**
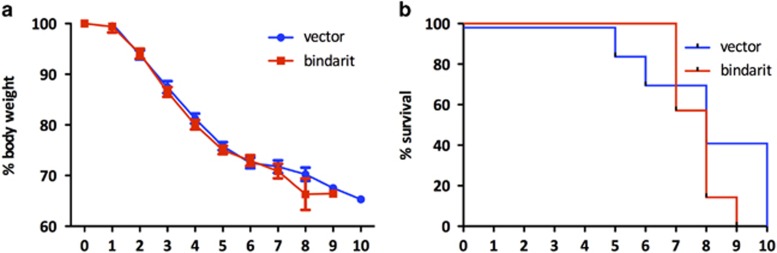
Effect of Bindarit treatment on weight loss and survival rate following avian IAV H7N9 infection. Mice were infected i.n. with a lethal dose (10 × LD_50_) of A/Anhui (H7N9). Mice were then treated with either Bindarit or vehicle starting at day 1 pi. Mice were monitored for weight loss (**a**) and survival (**b**). Time points represent days pi. Data are from seven mice per group±s.e.m.

The ability of Bindarit to protect mice from lethal H7N9 infection was investigated. Mice were intranasally (i.n.) infected with a lethal dose of H7N9 (10^5^ PFU) and treated with Bindarit (70 mg kg^−1^) twice daily starting at day 1 post-infection (pi). Mice were monitored for weight loss and survival. Bindarit treatment had no detectable impact on weight loss or survival of mice. Mice in both groups lost body weight to a similar extent, reaching a 25% reduction in body weight by day 5 (Figure 2a). Mortality typically occurred between day 5 and 10 pi for untreated mice and between days 7 and 9 for Bindarit-treated mice (Figure 2b). No significant (*P*<0.1) difference was noted in the survival rate between the two groups.

### Bindarit treatment does not affect lung pathology in mice infected with a lethal dose of avian IAV H7N9

**Figure 3:**
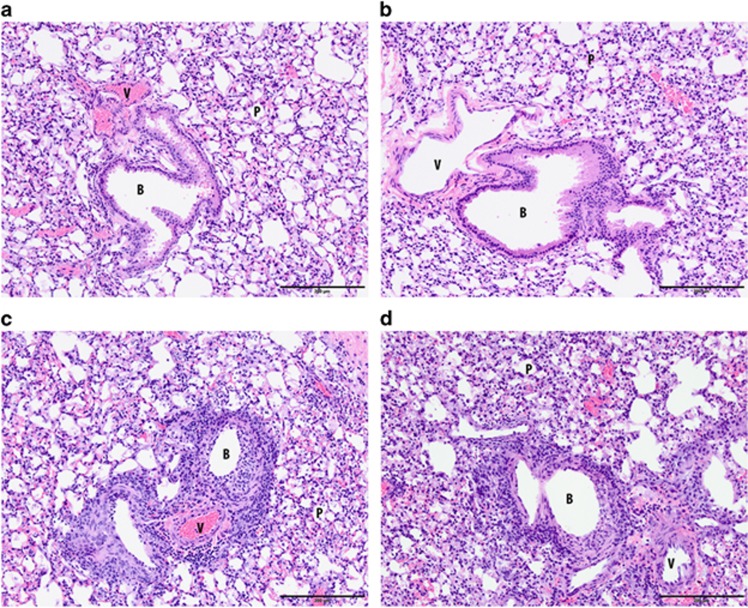
Effect of Bindarit on histopathological changes following avian IAV H7N9 infection. Mice were infected i.n. with a lethal dose (10 × LD_50_) of H7N9 or PBS. Mice were then treated with either Bindarit or vehicle starting at day 1 pi. At day 4 pi, mice were killed and lungs collected for histopathological analysis. Mock-infected methylcellulose (**a**) and Bindarit-treated (**b**). Viable epithelial cells line the bronchioles and there is no exudate within the lumen. No peribronchiolar or perivascular infiltrations are present and the parenchyma has no changes. H7N9-infected methylcellulose (**c**) and Bindarit-treated (**d**). There is diffuse necrosis and loss of the bronchiolar epithelium with luminal necrotic cellular debris lining the denuded wall. A mild to moderate peribronchiolar and perivascular infiltration of mostly lymphocytes is also present. Parenchymal changes are mild to moderate with mild thickening of the alveolar septa and a few inflammatory cells in alveoli. Data are from three mice per group. B, bronchiole; P, parenchyma; V, vessel.

The effect of Bindarit on lung inflammation and pathology was investigated in mice infected with H7N9. Mice were i.n. infected with a lethal dose of H7N9 (10^5^ PFU) and orally treated with Bindarit (70 mg kg^−1^) twice daily starting at day 1 pi. Mice were killed at day 4 pi and lungs were collected for histopathology. Lungs in both groups, mock- and Bindarit-treated, showed moderate to severe necrotic bronchitis and bronchiolitis. The peribronchiolar and perivascular infiltration was mild to moderate for both groups and animals showed mild to severe alveolitis (Figures 3c and d). Two mock-treated mice infected with H7N9 developed hemorrhage, a feature that was not observed in Bindarit-treated animals. Taken together, there were no substantial changes in histopathology when mice were orally treated with Bindarit (70 mg kg^−1^). All mice showed a lung score of 3 (Table 1).

### Bindarit treatment is associated with increased pulmonary cellular infiltration after lethal avian IAV H7N9 infection

**Figure 4:**
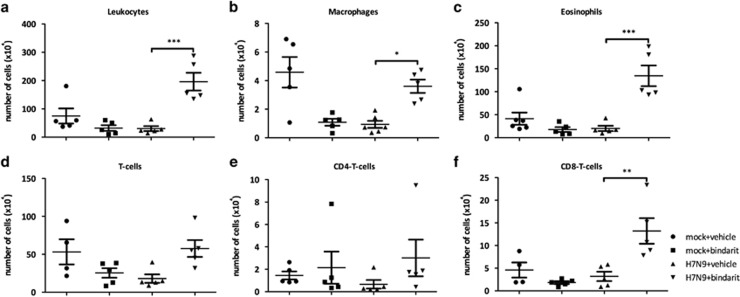
Effect of Bindarit on cellular infiltration following avian IAV H7N9 infection. Mice were infected i.n. with a lethal dose (10 × LD_50_) of A/Anhui (H7N9) or PBS. Mice were then treated with either Bindarit or vehicle starting at day 1 pi. At day 5 pi, mice were killed and BAL fluids were collected for analysis with flow cytometry. (**a**) The total number of BAL leukocytes, (**b**) the number of macrophages, (**c**) the number of eosinophils, (**d**) the number of CD3^+^ T-cells, (**e**) the number of CD4^+^ T cells, and (**f**) the number of CD8^+^ T cells were determined. Data are presented as the number of specific type of cells per million total cells. Data are from five mice per group±s.e.m. *P<0.05, **P<0.01, ***P<0.001.

To investigate the effects of Bindarit on pulmonary cellular infiltration, flow cytometry was performed using the bronchoalveolar lavage (BAL) fluid from infected mice. A significant (*P*<0.001) increase in total number of leukocytes was observed in mice orally treated with Bindarit (70 mg kg^−1^) after lethal infection with H7N9 at day 4 pi (Figure 4a). Interestingly, the number of alveolar macrophages was increased after Bindarit treatment (Figure 4b); despite its known ability to reduce production of the monocyte attractant factor MCP-1/CCL2. Furthermore, the influx of eosinophils and CD8+ T-cells was also increased in Bindarit-treated mice (Figures 4c and f).

### Bindarit treatment is associated with weight loss after sub-lethal infection with H7N9

**Figure 5:**
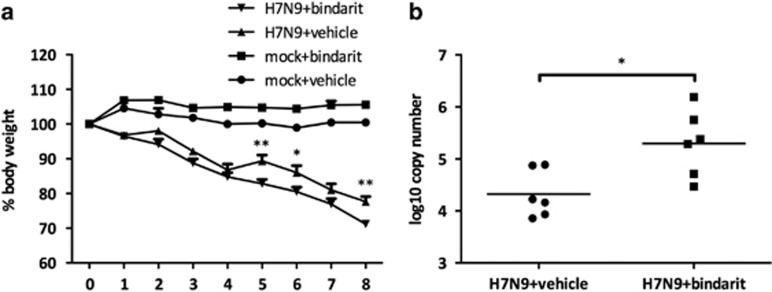
Effect of Bindarit treatment on weight loss and virus titer following avian IAV H7N9 infection. Mice were i.n. infected with a sub-lethal dose of H7N9 (10^2.7^ PFU) or PBS. Mice were then orally treated with either Bindarit or vehicle starting at day 1 pi. Mice were monitored for weight loss until day 8 pi (**a**), when mice were killed and lungs were collected for virus titer analysis with RT-qPCR (**b**). Virus titers were determined per 5 ng of total RNA extracted from lung homogenates. Time points represent days pi. Data are from five to six mice per group±s.e.m. *P<0.05, **P<0.01.

No differences in weight loss were observed between mock- and Bindarit-treated mice after i.n. lethal H7N9 infection (10^5^ PFU). The lethal challenge may have been overwhelming and therefore the effect of Bindarit insufficient. Therefore, a study using a sub-lethal dose of virus was performed. Mice were infected 10^2.7^ PFU with a sub-lethal dose of H7N9 and orally treated with Bindarit (70 mg kg^−1^) twice daily starting at day 1 pi. Mice in both groups lost body weight until day 4 pi. However, on days 5, 6 and 8, Bindarit-treated mice showed a considerable increase in weight loss compared to mock-treated control mice (Figure 5a).

### Bindarit treatment was associated with an increase in lung viral titers after sub-lethal H7N9 infection

In order to investigate the role of CCL2, the level of lung viral clearance was determined when mice were treated with Bindarit during H7N9 disease. Viral titers were measured by RT-qPCR from 5 ng of total RNA from homogenized lung samples using primers and probe specific to the viral M gene. Mice orally treated with Bindarit (70 mg kg^−1^) had higher lung virus titers compared to mock-treated control mice at day 8 pi (Figure 5b). The virus titer was ~1 log higher in the Bindarit-treated group.

### Bindarit treatment did not alter pro-inflammatory cytokines gene expression during sub-lethal H7N9 infection

**Figure 6:**
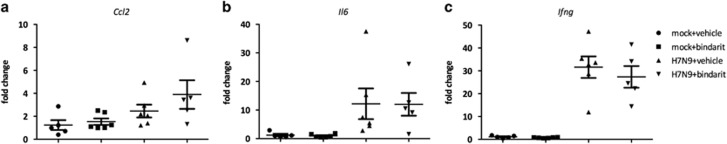
Effect of Bindarit on pro-inflammatory cytokine gene expression following avian IAV H7N9 infection. Mice were i.n. infected with a sub-lethal dose of H7N9 (10^2.7^ PFU) or PBS. Mice were then treated with either Bindarit or vehicle starting at day 1 pi. On day 8 pi, mice were killed and lungs were collected for gene expression analysis for *Ccl2* (**a**), *Il6* (**b**) and *Ifng* (**c**) with RT-qPCR. Expression was normalized to 18S and relative to non-infected mice. Data are from five mice per group±s.e.m. *P<0.05, **P<0.01.

The effect of Bindarit on the expression of pro-inflammatory cytokines in the lungs was investigated to show the effectiveness of oral Bindarit treatment. RT-qPCR was used to measure the expression of pro-inflammatory cytokine genes *Il6*, *Ifng* and *Ccl2* in the lungs at day 8 pi. *Il6* and *Ifng* were of particular interest as IL-6 has been linked to tissue destruction in mice infected with IAV,^17^ and IFNγ is known for its protective role against IAV disease.^32^ Bindarit did not appreciably modulate the level of these pro-inflammatory cytokines. The effect of oral Bindarit treatment on the expression of *Ccl2* and other cytokines in the lungs appeared to be minimal (Figure 6).

### Bindarit treatment affects pro-inflammatory cytokine levels after sub-lethal H7N9 infection

**Figure 7:**
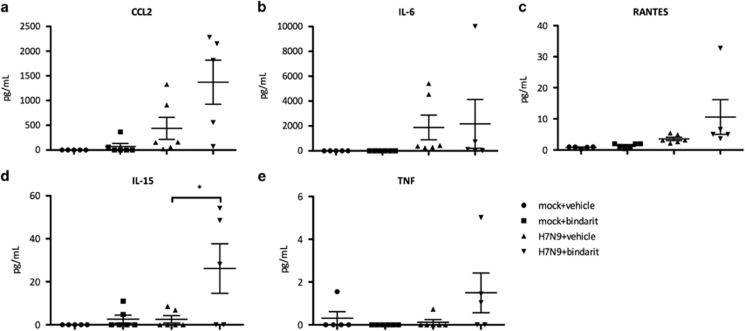
Effect of Bindarit on protein level of pro-inflammatory following avian IAV H7N9 infection. Mice were infected i.n. with a sub-lethal dose of A/Anhui (H7N9) or PBS. Mice were then treated with either Bindarit or vehicle starting at day 1 pi. On day 8 pi, mice were killed and BAL was collected for protein expression analysis of CCL2 (**a**), IL-6 (**b**), RANTES (**c**), IL-15 (**d**) and TNFα (**e**) with a multiplex enzyme-linked immunosorbent assay. Data are from five mice per group±s.e.m. *P<0.05.

To corroborate the RT-qPCR results, a Luminex enzyme-linked immunosorbent assay platform was used to measure the pro-inflammatory cytokines IL-6, IL-15, CCL2, RANTES and TNF in the BAL at day 8 pi (Figures 7a–e). The IL-15 protein level was significantly (*P*<0.05) increased when H7N9-infected mice were treated with Bindarit. Bindarit treatment did not appreciably change the level of other pro-inflammatory cytokines. Bindarit oral treatment also had minimal effect on cytokine expression levels in the lungs. Interestingly, CCL2 protein level was not reduced, but appeared enhanced after treatment with Bindarit.

### Bindarit treatment did not affect the pulmonary cellular influx during sub-lethal Avian AIV H7N9 infection

**Figure 8:**
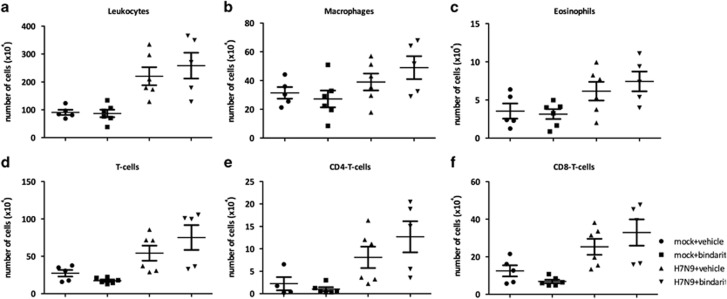
Effect of Bindarit on pulmonary cell infiltrates following avian IAV H7N9 infection. Mice were i.n. infected with a sub-lethal dose of H7N9 (10^2.7^ PFU) or PBS. Mice were then treated with either Bindarit or vehicle starting on day 1 pi. On day 8 pi mice were killed and BAL fluids collected for analysis with flow cytometry. (**a**) The total number of leukocytes, (**b**) the number of macrophages, (**c**) the number of eosinophils, (**d**) the number of CD3^+^ T-cells, (**e**) the number of CD4^+^ T cells, and (**f**) the number of CD8^+^ T cells were determined. Data are presented as the number of specific type of cells per million total cells. Data are from five mice per group±s.e.m. *P<0.05, **P>0.01, ***P>0.001.

To determine a mechanism for cellular infiltration into the BAL, the was investigated using flow cytometry. Total numbers of leukocytes as well as macrophages, eosinophils, and T cells were not significantly (*P*<0.01) increased after Bindarit treatment, but were slightly higher than those in mock-treated mice (Figure 8). The effect of Bindarit treatment on cellular infiltration appeared to be weaker after sub-lethal infection than after lethal infection. Interestingly, the number of alveolar macrophages did not change after treatment with Bindarit, despite its known ability to reduce production of monocyte attractant MCP-1/CCL2 in other models.^18^

## Discussion

The severe disease in mice infected with avian IAV H7N9 is associated with a ‘cytokine storm' characterized by upregulation of pro-inflammatory cytokines and chemokines such as CCL2, IL-6, IL-8, IFN-α, IFNγ, IP-10, MIG and macrophage inflammatory protein-1β. A similar cytokine response is thought to contribute to lung injury and severe pneumonia in H7N9-infected patients.^2^ Mouse studies suggested that TNFα and IFNγ may help to suppress viral gene expression and increase viral clearance, while CCL2 and IL-6 may contribute to lung injury during H7N9 disease.^17^ Therefore, this study aimed to investigate the role of CCL2 in the context of avian IAV H7N9 disease by using the potent CCL2 synthesis inhibitor Bindarit. Treatment of H7N9-infected mice with Bindarit may have enhanced some aspects of disease including increased virus titers, weight loss and cellular infiltration in the BAL. These results suggest that CCL2 has an antiviral role against H7N9 replication; thus, therapeutic approaches targeting CCL2 may be caution advised for the treatment of H7N9 influenza infection. This is the first study evaluating the effects of CCL2 inhibitors for the treatment of influenza-induced disease and the results suggest that this class of drugs may not be suitable for treatment of severe influenza infections.

CCL2 is upregulated in several viral diseases in humans, such as HIV, hepatitis C virus, several herpes viruses, Japanes encephalitis virus and respiratory syncytial virus, and has been considered as a biomarker linked to disease severity in HIV.^33^ Furthermore, CCL2 has been linked to inflammation and tissue damage in human disease.^33, 34, 35^ In animal studies of various inflammatory diseases, Bindarit was effective in reducing CCL2 production *in vitro* and *in vivo* and successfully alleviated CCL2-driven diseases such as arthritis, encephalomyelitis and prostate and breast cancers.^36, 37, 38^ In mouse models of arthritogenic alphavirus disease, Bindarit reduced disease symptoms such as clinical score, cellular infiltration of muscle tissues and bone loss but had no effect on virus clearance.^19, 20, 21^ These studies indicate that Bindarit may be potentially used in the treatment of virus-induced inflammation. Furthermore, Bindarit reduces inflammation and ameliorates disease in a mouse model of autoimmune encephalomyelitis,^37^ which mimics many aspects of Guillain–Barre syndrome,^39^ indicating that CCL2 inhibitors could potentially be beneficial in preventing exacerbated Guillain–Barre syndrome. However, CCL2 may have dual roles in antiviral defense, mediating both protective and pathogenic functions. For example, in a study evaluating the role of CCL2 using an animal model of HIV, CCL2 receptor (CCR2) knockout mice showed increased virus titers and disease.^35^ In a different study on CHIKV a similar importance for the CCR2 was found. CCR2 deficiency promoted exacerbated chronic erosive neutrophil dominated CHIKV-induced arthritis in mice.^40^

CCL2 is highly upregulated in patients suffering from H7N9 influenza infection, and has been linked to lung injury in mouse models.^2^ However, in this study, when H7N9-infected mice were treated with Bindarit, mice exhibited heightened disease signs as demonstrated by an increase in weight loss, pro-inflammatory factors, cellular infiltration and virus titers. Thus, blocking CCL2 dampened viral clearance and was associated with upregulation of pro-inflammatory cytokines and cellular infiltration. In earlier studies examining the effects of anti-CCL2 antibodies on IAV disease, mice exhibited enhanced pneumonitis compared to non-treated animals, despite reduced numbers of cellular infiltrates such as leukocytes, macrophages and neutrophils in the lungs.^41^ Furthermore, infection of CCL2 knockout mice with a non-lethal dose of a mouse-adapted strain of IAV resulted in a profound increase in weight loss, elevated viral loads and pro-inflammatory cytokines, and enhanced leukocyte recruitment into the infected lungs compared to wild-type mice.^42^ Interestingly, in that study, pro-inflammatory cytokines such as TNFα, IL-6 and IFNγ were enhanced, but cellular infiltrates into the lungs were reduced.^42^ However, one limitation of that study was the analysis of the cellular infiltrate in full lung homogenate as opposed to BAL, which may have influenced the outcome of the study. In the current study, we observed an increase in pro-inflammatory cytokine expression and cellular infiltration in the BAL.

Interestingly, *Ccl2* gene expression was not reduced in the lungs after oral treatment with Bindarit, despite its known capability in reducing CCL2 synthesis *in vitro* and *in vivo* from earlier studies by other groups.^18^ Oral administration of Bindarit may be a limitation in the treatment of pneumonia, as there are difficulties in reaching therapeutic concentrations of drugs in the lungs when administered by this route.^43^ It remains a possibility that oral delivery of Bindarit was not completely effective or led to a systemic reduction of CCL2 production that contributed to disease enhancement. In future studies this limitation will be addressed by i.n. administration of Bindarit, which may increase the concentration of the drug in the lungs. In addition, IL-15 was highly upregulated in the BAL of Bindarit-treated H7N9-infected mice. IL-15 has recently been described as a critical factor in the pathogenesis of IAV in mice with virus-induced acute lung injury.^44^ Whether there is a link between increased IL-15 production in the lung and a systemic inhibition of CCL2 synthesis remains a subject of further studies.

Various approaches have been investigated for the treatment of H7N9 infection in the recent years. Treatment with corticosteroids was evaluated, but it led to increased mortality in patients suffering from acute H7N9 infection.^45^ Due to increased drug resistance among circulating and novel IAV strains and the lack of specific vaccines against H7N9, there remains an imminent need for drug repurposing because the development of novel antivirals and specific vaccines will require many years of preclinical and clinical studies before their availability for clinical use.

## Methods

### Cell cultures and influenza virus stock

All *in vivo* experiments were performed under the guidance of the Institutional Animal Care and Use Committee (IACUC) and Animal Resources at the Animal Health Research Center (AHRC), which has approved biosafety level 3 laboratories. Avian IAV A/Anhui/1/2013 (A/Anhui; H7N9) was propagated in embryonated chicken eggs. Influenza A/California/04/09 (A/Ca; H1N1) was propagated in Madin-Darby canine kidney cells (ATCC CCL-34). Viruses were titrated on Madin-Darby canine kidney cells as described previously.^46^ The human type II respiratory epithelial cell line A549 (ATCC CCL-185) was maintained in Dulbecco's modified eagle's medium (HyClone, Logan, UT, USA) supplemented with 5% heat-inactivated fetal bovine serum (HyClone) in a 37 °C incubator with 5% CO_2_. Cell lines were regularly tested for mycoplasma contamination. Virus propagation in embryonated eggs was carried out in strict accordance with the recommendations by the University of Georgia IACUC. The protocol was approved by the University of Georgia IACUC.

### *In vitro* influenza infection and Bindarit treatment

Bindarit (2-Methyl-2-[[1-(phenylmethyl)-1H-indazol-3-yl]methoxy]propanoic acid) was synthesized by Chemlin (Nanjing, China). Bindarit was dissolved in ultrapure water (Thermo Fisher Scientific, Waltham, MA, USA). A549 cells were grown to 80% confluency in a 48-well plate and infected with A/Ca (H1N1) at multiplicity of infection (MOI) of 0.1. After infection, Bindarit was added to the wells at a concentration of 100 μm. After 24 h, cells were collected in TRIzol (Thermo Fisher) for total RNA purification. Gene expression was assessed using RT-qPCR. All infections were performed in the presence of 1 μg ml^−1^ (l-tosylamido-2-phenyl) ethyl chloro-methyl ketone (TPCK)-treated trypsin (Worthington Biochemical, Lakewood, NJ, USA) in modified Eagle's medium (MEM) supplemented with 0.3% bovine serum albumin (Thermo Fisher Scientific).

### *In vivo* influenza infection and Bindarit treatment

BALB/c female mice (6-to-8 weeks old) were obtained from the National Cancer Institute. All experiments and procedures were approved by the Institutional Animal Care and Use Committee (IACUC) of the University of Georgia. All experiments were performed with 5–7 mice per group and repeated independently at least twice. Bindarit was dissolved in 0.5% methylcellulose (Thermo Fisher Scientific) at 7 mg ml^−1^ (maximum solubility). To evaluate the effect of Bindarit on disease burden, Bindarit was administered using 0.2 ml (equivalent to 70 mg kg^−1^) oral gavage twice daily starting at day 1 pi. Mice were inoculated i.n. with influenza virus strain A/Anhui/1/13 (H7N9) with either a lethal dose (10^5^ PFU) or a sub-lethal dose (10^2.7^ PFU) of H7N9. BAL fluid was collected in phosphate-buffered saline (PBS) to determine cell number with flow cytometry and cytokine levels using multiplex enzyme-linked immunosorbent assay. Lungs were collected in 10% buffered formalin for histopathological analyses or homogenized in serum-free Dulbecco's modified eagle's medium and processed with TRIzol for RNA extraction and subsequent RT-qPCR.

### RNA isolation and RT-qPCR

Total RNA was isolated using TRIzol as previously described.^47^ In brief, the concentration of total RNA was measured using a microplate spectrophotometer (Epoch; BioTek, Winooski, VT, USA). RT-qPCR was used to validate mRNA expression changes and virus load using the Stratagene Mx3005P real-time PCR system (Agilent Technologies, Santa Clara, CA, USA). The reverse transcription reactions were performed using the SuperScript VILO cDNA Synthesis Kit (Thermo Fisher Scientific) and 1000 ng total RNA for each reaction. qPCR was performed using the GoTaq Green Master Mix (Promega, Madison, WI, USA) to determine mRNA levels, and data were normalized to 18S expression using the 2^−ΔΔCt^ method.^48^ Primer sequences for *CCL2* were: 5′-GAACACACTCAGCGCAGTTA-3′ (forward primer) and 5′-CACCCACCCTCTCTTTGATTAC-3′ (reverse primer). The virus load was determined using 5 ng of total RNA with a TaqMan Fast Virus 1-Step Master Mix (Thermo Fisher Scientific). The standard curve was produced using an M gene plasmid.^49^

### Histopathological evaluation

Lungs from infected mice were harvested at 4 days (pi), perfused with 10% buffered formalin, and fixed in 10% buffered formalin. The sections were embedded in paraffin, cut into 5 μm sections, and stained with hematoxylin and eosin. The sections were evaluated using light microscopy. A histological score for each lung was determined according to the following criteria: 0=no lung abnormality; 1=<10% of airways inflamed; 2=10–30% of airways inflamed; 3=30–50% of airways inflamed and 4=>50% of airways inflamed.^50^ The slides were evaluated by a pathologist without prior knowledge of the infection and treatment status.

### BAL collection and quantification of cytokines

Eight days pi, mice were killed and tracheotomy was performed. The mouse lungs were flushed with 1 ml of PBS, and the retained BAL was centrifuged at 400 *g* for 5 min at 4 °C. The recovered supernatants were collected and stored at −80 °C until assessed for cytokine concentration, and the cell pellet were resuspended in 200 μl of 10% buffered formalin. Total cell numbers were counted using a hemocytometer. Cytokines in BAL supernatants were quantified with the Luminex xMAP system using a MILLIPLEX MAP mouse cytokine immunoassay (MCYTOMAG-70K; Millipore, Billerica, CA, USA) according to the manufacturer protocol. In brief, beads coupled with anti-CCL2, anti-IL-6, anti-interferon-γ, anti-RANTES, anti-IL-15 and anti-TNF monoclonal antibodies were sonicated, mixed and diluted 1:50 in assay buffer. For the assay, 25 μl of beads were mixed with 25 μl of PBS, 25 μl of assay buffer, and 25 μl of BAL supernatant, and incubated overnight at 4 °C. After washing, beads were incubated with biotinylated detection antibodies for 1 h and the reaction mixture was then incubated with streptavidin-phycoerythrin (PE) conjugate for 30 min at room temperature, washed and resuspended in PBS. The assay was analyzed on a Luminex 200 instrument (Luminex Corporation, Austin, TX, USA) using the Luminex xPONENT 3.1 software.

### Flow cytometry

For flow cytometry analysis, cell suspensions were incubated in FACS staining buffer (PBS containing 1% BSA) and subsequently stained for 30 min at 4 °C with an optimized concentration of antibodies (BD Bioscience, Franklin Lakes, NJ, USA): PE-conjugated anti-CD3, PerCPCy5.5-conjugated anti-CD8, PE Cy7-conjugated anti-CD4, PerCPCy5.5-conjugated anti-CD45, APC-conjugated anti-CD11c and PE-conjugated anti-SiglecF to determine cell types in the BAL. Cells were acquired on an LSRII flow cytometer (BD Bioscience) and the data were analyzed using the FlowJo software (v 7.6.5; Ashland, OR, USA). Based on surface marker expression, six different cell types were identified: CD45^+^ (total leukocytes), CD45^+^SiglecF^+^CD11c^low^ (eosinophils), CD45^+^SiglecF^+^CD11c^high^ (alveolar macrophages), CD45^+^CD3^+^ (total T cells), CD4 T cells (CD45^+^CD3^+^CD4^+^) and CD8 T cells (CD45^+^CD3^+^CD8^+^).

### Statistical analysis

All experiments were performed in minimum in triplicate to ensure adequate power and the experiment independently repeated at least twice. Using power analysis, 12 animals in each group are required to give a 90% probability of detecting a treatment difference at a 5% significance level if the true difference between the treatments is one s.d. of the variation with each treatment group. However, decreasing the number of mice to five per group, gives a 90% probability of detecting a treatment difference if the true difference between the treatments is 1.63 s.d.'s of the variation with each treatment group. Further decreasing the treatment group size to three mice per group, gives a 90% probability of detecting a treatment difference only if the true difference between the treatments is 2.36 s.d.'s of the variation with each treatment group. Data are expressed as mean±s.e.m. Differences between groups were determined by one-way analysis of variance. Individual differences between groups were tested by multiple comparison and analysis using the Tukey post-test. Pairs of groups were compared by Student's *t*-test (two tailed). *P*-values of <0.05 were considered significant. All analysis was performed using Graphpad Prism Software (La Jolla, CA, USA).

## Figures and Tables

**Table 1:** Lung scores and observations of lesions after lethal H7N9 infection in mice

*Group*	*Lung score*	*Bronchitis/bronchiolitis*	*Peribronchial/perivascular infiltration*	*Alveolitis*	*Additional observation*
H7N9+vehicle	3	Severe necrosis	Moderate	Mild	
	3	Moderate necrosis	Mild	Moderate	
	3	Moderate necrosis	Mild	Severe	Hemorrhage
	3	Severe necrosis	Moderate	Moderate	Hemorrhage
H7N9+Bindarit	3	Moderate necrosis	Moderate	Moderate	
	3	Moderate necrosis	Moderate	Moderate	
	3	Moderate necrosis	Moderate	Moderate	
